# Ultrasonication-Tailored Graphene Oxide of Varying Sizes in Multiple-Equilibrium-Route-Enhanced Adsorption for Aqueous Removal of Acridine Orange

**DOI:** 10.3390/molecules28104179

**Published:** 2023-05-18

**Authors:** Zhaoyang Han, Ling Sun, Yingying Chu, Jing Wang, Chenyu Wei, Yifang Liu, Qianlei Jiang, Changbao Han, Hui Yan, Xuemei Song

**Affiliations:** 1 Key Laboratory of Advanced Functional Materials, Institute of Advanced Energy Materials and Devices, Ministry of Education, Faculty of Materials and Manufacturing, Beijing University of Technology, Beijing 100124, China; 2 Beijing Guyue New Materials Research Institute, Beijing University of Technology, Beijing 100124, China

**Keywords:** multiple-equilibrium route, graphene oxide, enhanced adsorption, acridine orange, size effect

## Abstract

Graphene oxide (GO) has shown remarkable performance in the multiple-equilibrium-route adsorption (MER) process, which is characterized by further activation of GO through an in-situ reduction process based on single-equilibrium-route adsorption (SER), generating new adsorption sites and achieving an adsorption capacity increase. However, the effect of GO on MER adsorption in lateral size and thickness is still unclear. Here, GO sheets were sonicated for different lengths of time, and the adsorption of MER and SER was investigated at three temperatures to remove the typical cationic dye, acridine orange (AO). After sonication, we found that freshly prepared GO was greatly reduced in lateral size and thickness. In about 30 min, the thickness of GO decreased dramatically from several atomic layers to fewer atomic layers to a single atomic layer, which was completely stripped off; after that, the monolayer lateral size reduction dominated until it remained constant. Surface functional sites, such as hydroxyl groups, showed little change in the experiments. However, GO mainly reduces the C=O and C-O bonds in MER, except for the conjugated carbon backbone (C-C). The SER adsorption kinetics of all temperatures fitted the pseudo-first-order and pseudo-second-order models, yet room temperature preferred the latter. An overall adsorption enhancement appeared as sonication time, but the equilibrium capacity of SER GO generally increased with thickness and decreased with the single-layer lateral size, while MER GO conversed concerning the thickness. The escalated temperature facilitated the exfoliation of GO regarding the adsorption mechanism. Thus, the isotherm behaviors of the SER GO changed from the Freundlich model to Langmuir as size and temperature changed, while the MER GO were all of the Freundlich. A record capacity of ~4.3 g of AO per gram of GO was obtained from the MER adsorption with a sixty-minute ultrasonicated GO at 313.15 K. This work promises a cornerstone for MER adsorption with GO as an adsorbent.

## 1. Introduction

With the development of society, organic dyes are widely used in leather, rubber, textile, papermaking, and food industries. Much of this effluent is released into the environment without treatment, leading to problems with water pollution [1,2,3,4]. Degradation of organic dye effluents is difficult and expensive due to four significant problems to overcome: an enormous discharge scale, high levels of nitrogen and phosphorus, a deep color of alkaline wastewater, and frequent changes in water quality. Thus, it has been long urgent to take adequate measures. Methods of treatment have been developed, including flocculation sedimentation [5], the ion exchange method [6,7], membrane separation [8], chemical oxidation [9], biological processing [10], and adsorption [11]. These methods have unignorable shortcomings in common, including complex operations, high costs, and potential ecological risks regarding additional treatments [1]. It is noteworthy that adsorption is more of a choice due to the advantages of the simple process and relatively low cost [12].

Conventional sorption follows a typical mixing and equilibrating fashion (reported as the single-equilibrium route, SER) with only one equilibrium throughout the process [13,14,15,16,17]. Regarding this fashion, many adsorbents have been investigated [14,15,18,19,20]. As is well known, graphene oxide (GO) is intrinsically the typical potent adsorbent in such a conventional way (acridine orange, AO, by a monodispersed GO sheet, the maximum reported ~1400 mg/g [21]; methylene blue, MB, 1939 mg/g [2]; and methyl orange, 138.96 mg/g [22]). It is often prepared from graphite by either the Hummers, Staudenmaier, or Brodie method [23,24,25,26,27]. The exfoliated raw two-dimensional graphene sheet carries many oxygen-containing groups, such as epoxy, carbonyl, hydroxyl, and carboxyl [24,25,28]. Therefore, GO can adsorb organic dyes through electrostatic, hydrogen-bonding, and π–π interactions [29].

As compared, the multiple-equilibrium route (MER) is an emerging and effective way to enhance adsorption further [30]. It features multiple equilibriums sequentially occurring throughout a process to best utilize the potentially available active functional sites across the adsorbent structure. Currently, MER is implemented by an in situ reduction [30,31]. It mainly includes two stages. In the first stage, GO was adsorbed in an acridine orange (AO) solution to form GO/AO composites. When the first adsorption stage reached equilibrium, a Na_2_S_2_O_4_ solution was added to the mixed solution of GO and AO. The second stage is when Na_2_S_2_O_4_ acts on the GO/AO composite, which increases the active adsorption sites on the surface and further adsorbs AO. It is noteworthy that Na_2_S_2_O_4_ has almost no effect on the structure of AO. Through MER, GO demonstrated excellent adsorption properties. For AO, the adsorption capacity of GO was increased more than twice as 3300 mg/g per by an in situ reduction with the droplet solution of Na_2_S_2_O_4_ [30,32]. The key to such an in situ reduction was converting the GO carbonyl group to a hydroxyl group, which increased the active working sites [30]. Altering the reducing system, Hao et al. [33] adopted the MER using NH_4_Cl to catalyze the reduction of GO by Zn powder and succeeded in the extreme adsorption of organic dyes (MB, 2600 mg/g; congo red, 7600 mg/g; and lemon yellow, 3200 mg/g). The above two studies indicate that the in situ reduction method can significantly improve the adsorption capacity of GO. Therefore, the structure potential of GO received further mining.

The oxygen-containing groups play an essential role in the adsorption of pollutants [21,34,35,36]. The C/O ratio of GO with different sizes showed noticeable differences [37,38,39,40]. Previous studies have shown that, as GO sheet size differs, the properties of surface oxygen-containing groups and adsorption capacity vary [36,41,42,43,44]. For instance, Ji et al. [26] found that the surface chemistry, structures, and supramolecular behaviors of GO strongly depend on their sizes. Zhang et al. [36] reported that aerogels made of Large GO (>20 μm) showed better absorptivity than those of crude GO, Mid-size GO (2–20 μm), and Small GO (<2 μm) precursors. Shen et al. [44] established the 3D GBMs from the largest GO nanosheets (20 μm versus 1 μm, 5–8 μm) exhibiting the fastest adsorption rate for MB removal. Therefore, it is necessary to emphasize the effect of particle size on the adsorption properties when performing MER. If so, the adsorption capacity of GO can be understood in greater depth. Realizing the high-efficiency use of GO and unveiling the inside mechanism shall benefit global water recycling. Moreover, it facilitates satisfying global carbon emission reduction targets.

Currently, many methods are available for sizing GO, including oxidation-controlling preparation [45,46], centrifugal separation [47], pH adjustment precipitation [42], time/power-confined ultrasonication [48], and extraction and filtration [43]. In this work, ultrasonication was adopted for simplicity and ease of control. To unveil the size effect of GO in the MER adsorption, we used temperature-constant water-bath ultrasonication to shear cut the GO. Afterward, GO was investigated on the condition that both SER- and MER-modeled adsorption with the cationic dye AO as an eliminating target was carried out and compared. As a result, differently sized GO in the SER and MER adsorption performed quite differently from each other. The mechanism was discussed from a perspective of difference regarding not only the lateral dimension but also the vertical layers and harnessed exterior temperature. Most importantly, GO with ever higher capacity of adsorption was observed.

## 2. Results

### 2.1. Sonicated Graphene Oxide and Acridine Orange

**Figure 1:**
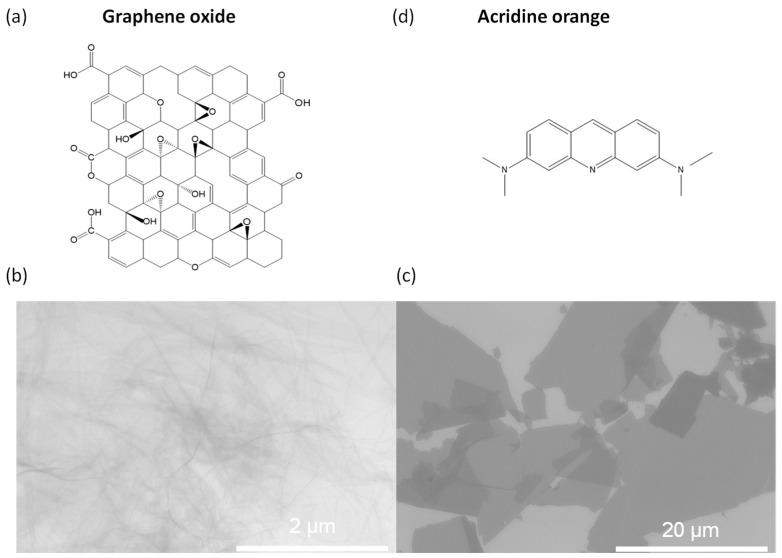
Schematic diagram of the chemical structure of (**a**) GO (graphene oxide), and (**d**) acridine orange and the freshly prepared GO sheets observed using the TEM (**b**) and SEM (**c**).

GO was used as an adsorbent in the present work. Figure 1a shows the typical chemical structure of GO [49], with a large variety of oxygen-containing groups on the 2D planar carbon skeleton. These groups are potentially active sites to bridge the GO sheet and alien ions/molecules [50,51]. Graphite was used as the feedstock for the preparation of graphene oxide in this work, following the modified Hummer method [52,53,54]. The preparation success was concluded from the typical scanning and transmission observation of GO. The morphology of GO presented many wrinkles on the GO surfaces (Figure 1b) and a character of dispersive distribution of sizes (Figure 1c), consistent with previous studies [30,41,55]. The lateral size of the rough GO sheets may exceed tens of microns, the same size as the original graphite, and will also be in the order of a few microns, indicating the effectiveness of ultrasonic treatment during the process. Ultrasound can reasonably be chosen to prepare GOs of different sizes, and proper timing is crucial.

**Figure 2:**
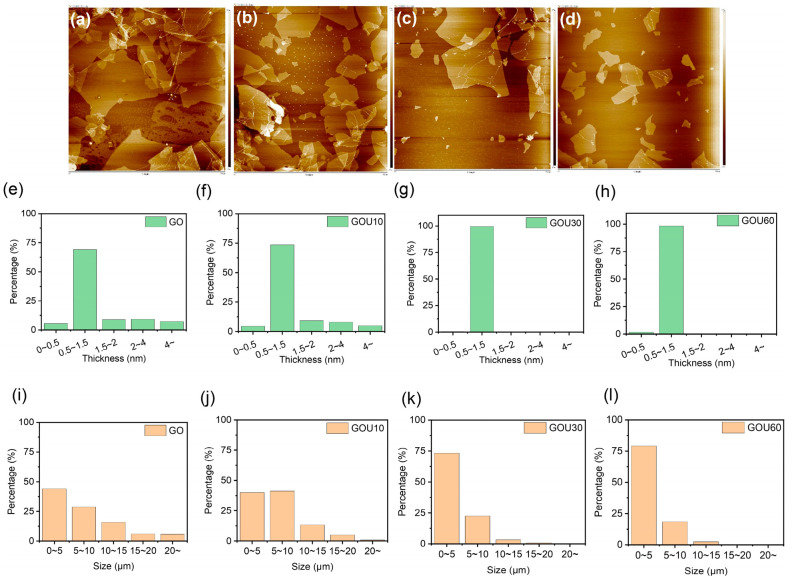
(**a**–**d**) Size variation of GO (graphene oxide) nanosheets with a concentration of 0.9 mg/mL as ultrasonication time (frequency of 27 KHz, 100 Watt) in a water bath with a temperature of 40 °C Note: the crude GO with the distribution of thickness (**e**) and lateral sizes (**i**); GOU10: ten-minute sonicated GO, (**f**,**j**); GOU30: thirty-minute sonicated GO (**g**,**k**); GOU60: sixty-minute sonicated GO (**h**,**l**).

In this study, sonication times were 10 min (GOU10), 30 min (GOU30), and 60 min (GOU60) because, after 60 min, no significant size changes were found compared to GOU60 (Figure 2a–d). More details about the thickness and lateral size of GO, GOU10, GOU30, and GOU60 were gathered in Figure 2. In the top group images of Figure 2, the AFM delivered a visible change regarding the thickness and lateral size from GO through GOU10 and GOU30 to GOU60. As reported [42,44], the thickness of single-layer GO often ranges from 0.6 to 1.2 nm, so there was a certain amount of multilayer GO for GO and GOU10. The account of single-layer GO sheets varied from around 75% to nearly 100% and the lateral dimension of over 5 μm from about 50% to less than 25% (Figure 2b). This indicates that the lateral size and thickness of GO decrease significantly with the increase of ultrasound time. After 30 min, the lateral size and thickness distribution of GO became stable (GOU30: lateral size 0~15 μm, 99.2%; thickness 99%. GOU30: lateral size 0~15 μm, 100%; thickness 100%.)

Acridine orange, previously selected as the decontaminating target [30], continued to play the same role. First, AO is tough to degrade and can resist changing conditions we made [56], becoming the typical dye we consider the adsorbate. Second, large amounts of cationic dye (Figure 1d) can be adsorbed to electronegative GO. Third, the in situ reduction process had little influence on the structure of acridine orange. Therefore, it is appropriate to explore the adsorption potential of GO using AO.

### 2.2. Comparative Adsorption of the MER and SER Involving Size-Different GO

**Figure 3:**
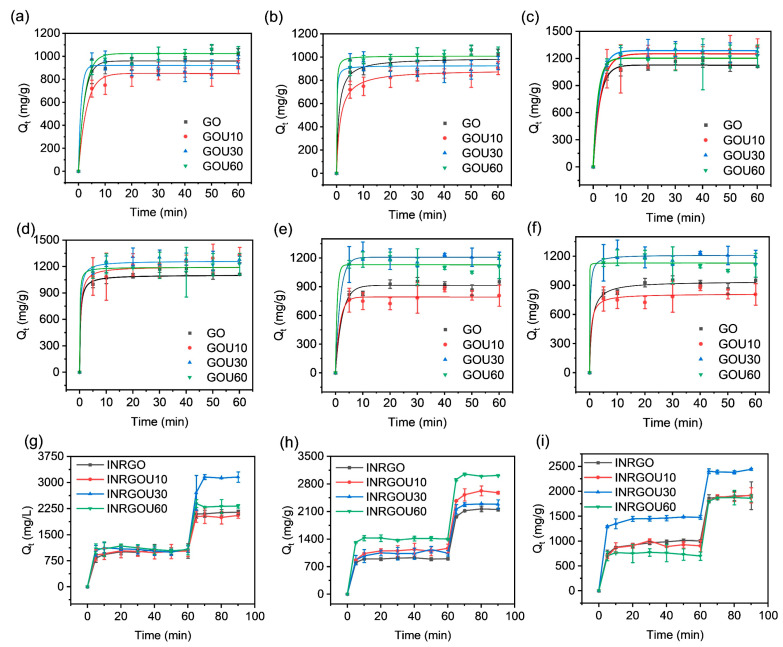
Time-dependent adsorption of GOs (graphene oxide samples): CG (control group) (**a**,**c**,**e**) at 10, 25, and 40 °C with the pseudo-first-order (PFO) model fitting; CG (**b**,**d**,**f**) at 10, 25, and 40 °C with the pseudo-second-order (PSO) model fitting; and EG (**g**,**h**,**i**) at 10, 25, and 40 °C without model fitting. The initial concentration of AO, 100 mg/L,20 mL; GO, 0.9 mg/mL, 0.2 mL.

Adsorption experiments with the above GO were conducted at 10, 25, and 40 °C (Figure 3). As shown, the adsorbing capacity of all GOs increased rapidly as time passed until there was no change. The SER process for control group (CG) and the MER process for experimental group (EG) reached equilibrium within 5 min and 10 min, respectively, indicating a fast, strong interaction between GO and AO. The efficient adsorption of CG and EG is promising in wastewater treatment. MER showed a typical double-equilibrium character, resulting in a significant increase in the adsorption capacity of EG (EG_max_: 3158.15 mg/g; CG_max_: 1331.85 mg/g).

All SER results except MER were treated by model fitting. With those fittings (Figure 3a–f and Table S1), the behavior of the CG with different sizes is found to correlate with both the pseudo-first-order (PFO) and pseudo-second-order (PSO) models (R^2^ > 0.96). Among these adsorbents, adsorption at room temperature (25 °C) is more consistent with the PFO model. In the CG group, adsorption at 10 °C and 40 °C appeared to be more in line with the PFO model for GOU30 and GOU60. This result reflects that the rate was proportional to the square of the unoccupied number of adsorptive sites [31]. The results show adsorption based on van der Waals forces, π–π interactions, and hydrogen bonds predominate in adsorption. Therefore, the adsorption behavior of GO can be affected by temperature.

Table S1 also shows the theoretical equilibrium capacity of CG, which is kept around 1000 micrograms per gram of GO and up to a maximum of 1287.00 mg/g (GOU30). This result highly accords with our decade-ago report (~1255 mg/g) [30], which confirms that GO made as such had an excellent reproducibility of their performance. Compared with the CG, the adsorption capacity of the EG (Figure 3g–i) was significantly increased. This change was identical to the previous results [30,33].

When comparing the adsorption of CG and EG, the particle size difference affects the amount of GO adsorbed, but this effect varies with conditions such as temperature, initial concentration, and the route of adsorption. The initial AO concentration was around 100 mg/mL, and the adsorptions applied both the SER and MER. At the same temperature, the individual GO capacity leveled up first and then fell as the size decreased—a common trend for most CG and EG samples. This trend accounted for 66.7% of SER samples, 33.3% of MER samples, and 50% of all samples. Overall, however, the adsorption capacity of GOs treated with sonication for either 30 min (GOU30) or 60 min (GOU60) was increased relative to the raw GO. This is expected due to the smaller size and larger surface area of GOU30 or GOU60, resulting in more adsorbable sites. Such enhancement came out somewhat differently at different temperatures, significantly if the adsorption route was changed. At a low temperature of 10 °C, the SER of GO sonicated for 60 min (GOU60) increased by 38.52 mg/g compared with the raw GO, while the gap widened to 116.67 mg/g at 25 °C. The novel MER widened these gaps to 1012.96 mg/g at 10 °C and 864.08 mg/g at 25 °C, almost by orders of magnitude (Table S1).

The reaction rate constant (K_1_, K_2_) was investigated and compared since the CG adsorptions correlated well with the PFO and PSO kinetics [57,58]. The adsorption rate constant positively relates to the adsorption rate [43]. We then found that the smaller the size of GO, the faster the reaction rate. At 40 °C, the smallest GO (GOU60) was of the highest chemical reaction rate (Figure S1).

Figure S3 illustrates the correlation statistics between sonication time and the adsorption capacity. The adsorption capacity was assumed as a function of sonication time. As a result, a tendency to increase was evident wherever for the CG (Figure S3a) or EG (Figure S3b).

**Figure 4:**
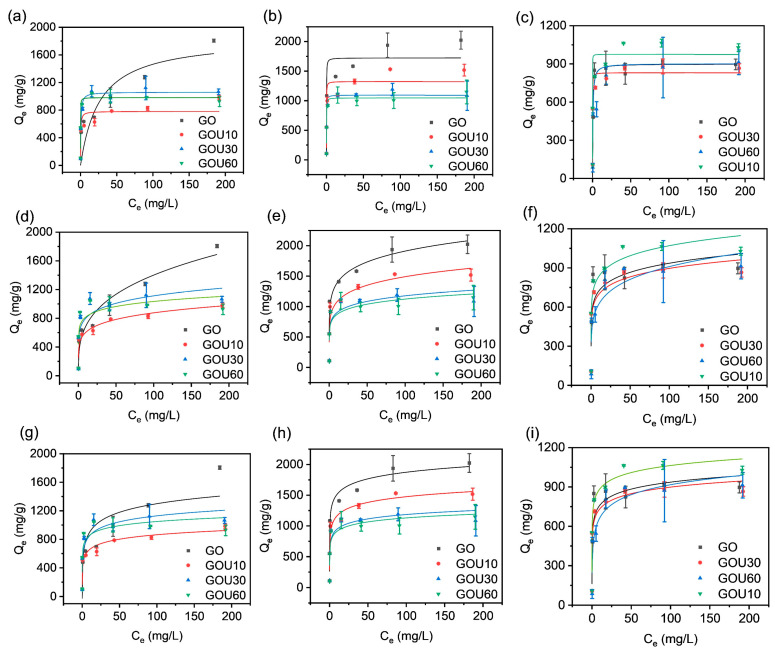
Isotherm adsorption of the control group (CG) with the Langmuir, Freundlich, and Tempkin model fitting: (**a**,**d**,**g**) at 10 °C; (**b**,**e**,**h**) at 25 °C; and (**c**,**f**,**i**) at 40 °C. The added volume of graphene oxide (GO) was 0.2 mL with a concentration of 0.9 mg/mL. Initial AO concentrations were 1, 5, 10, 25, 50, 100, and 200 mg/L.

To explore the isotherm behavior, the isotherm experiments were conducted with different initial concentrations regarding the temperature, sheet size, and adsorption route applied. The results involving the fittings of CG are shown in Figure 4 and Table S3. With the increase of initial concentration, the solution’s final equilibrium concentration increased, while CG’s experimental equilibrium capacity increased rapidly at first and then slowly. The maximum capacity of individual CG did not always increase with temperature since higher temperatures (25 °C) reversely limited the adsorption capacity of CG (Table S3).

However, at the same temperature, the typical finding revealed that the sonicated GO fell behind the untreated GO except for at 40 °C. That is, at 10 and 25 °C, the capacity of sonicated GO was higher than that of GOU10, GOU30, and GOU60, being 1806.67 mg/g and 2025.19 mg/g, respectively. Such excellence at the lower temperature may be attributed to many fluffy-multilayer structures of GO that allowed AO to enter the interlayers, thereby increasing the adsorption capacity. The decrease in the adsorption performance of GO at 40 °C should be due to the flaking of the fluffy-multilayer structure into a single layer of GO under high-temperature and vibration conditions. Furthermore, previous studies have reported that the multilayer structure could change the adsorption behavior [36,59,60].

The adsorption of GO was higher than that of GOU10 at 10 and 25 °C. Interestingly, at 40 °C, the adsorption capacity of sonicated GO, especially the GOU10 (1644.44 mg/g), was reversely beyond that of GO. It means the capacity positively correlated with the GO size if the temperature was not that high, as also observed statistically from the kinetics.

Accordingly, at lower temperatures, less sonicated GO (e.g., GO and GOU10) behaved in the Freundlich and Tempkin models and not in the Langmuir, but as the temperature was high, GO and GOU10 turned out to be of the Langmuir monolayer adsorption (Tables S3 and S6). As discussed above, this difference was possible because the multilayer structure was gradually bankrupted into single sheets as the temperature rose (single-layer GO > 99%, Figure 2), and simultaneously the adsorption mechanism changed to monolayer adsorption. Due to the same reason, the GOU30 and GOU60 conformed to the Langmuir model at all temperatures.

**Figure 5:**
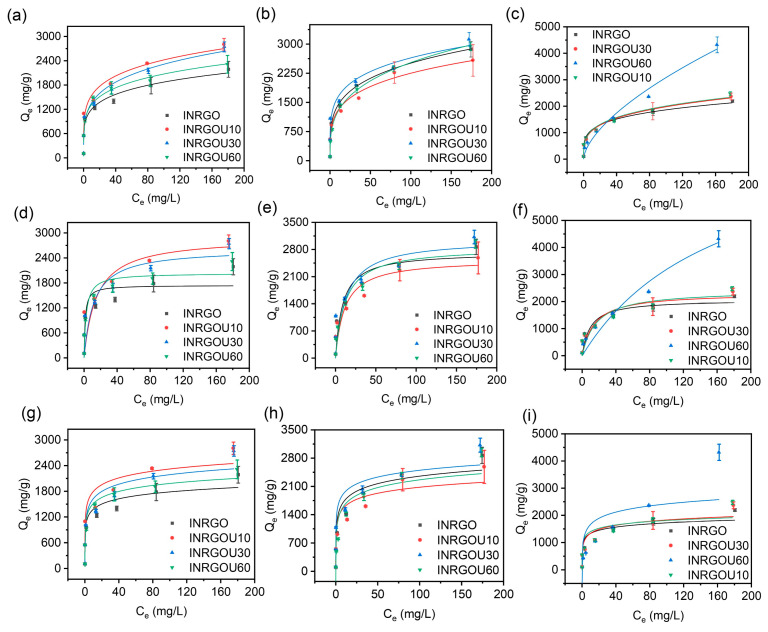
Isotherm adsorption of the experimental group (EG) with the Langmuir, Freundlich, and Tempkin model fitting: (**a**,**d**,**g**) at 10 °C; (**b**,**e**,**h**) at 25 °C; and (**c**,**f**,**i**) at 40 °C. The added volume of graphene oxide (GO) was 0.2 mL with a concentration of 0.9 mg/mL. Initial AO concentrations were 1, 5, 10, 25, 50, 100, and 200 mg/L.

Figure 5 shows the isotherm adsorption results of EG. It can be found that the equilibrium adsorption amount of EG gradually increases with the increase of the equilibrium concentration of AO.

However, the sample of EG did not reach the trend of saturation of adsorption capacity. It was reflected in the fittings (Table S4). For EG, such MER-pathing adsorptions were all of the Freundlich behavior, other than the Langmuir or Tempkin. This fitting ascertained that the adsorptions were all a physical interaction dominating the process. Therefore, the adsorption capacity of AO by EG was significantly better than that of CG. For EG and CG, the GO adsorption capacity per gram is several thousand milligrams. For example, at 40 °C with an initial AO concentration of 200 mg/g, the capacity was 4322.96 mg/g for EG, while for CG, it was only 1028.15 mg/g.

From the global perspective, the EG showed the maximum capacity reduced when the temperature increased. For example, the maximum adsorption capacities for the INRGOU10 reached 2808.15, 286.67, and 2481.48 mg/g at 10, 25, and 40 °C, respectively. The EG received the maximum adsorption capacity up to 4322.96 mg/g, derived from the INRGOU60 at 40 °C. As reported elsewhere, the isothermal behavior of GO conversion to Freundlich after the addition of excess Na_2_S_2_O_4_ (0.2 g/mL, 0.02 mL) should be the main reason for the sharp increase in GO [30].

Moreover, we also observed that the maximum capacity increased as the sizes of GO sheets became smaller by sonication. The INRGOU60 defines the GO as being firstly temperature-constantly sonicated for 60 min, theoretically taking on the fewest particle sizes as compared, and then used and sampled through the MER process. As a result (Table S4), it resulted in the largest capacity, just like the speculation of size effect mentioned in the kinetic analysis, possibly due to more functioning groups on the enough-sufficient surfaces by the in situ reduction, MER. To further investigate how the MER affected the adsorption properties of EG, the following characterizations continued.

## 3. Discussion

**Figure 6:**
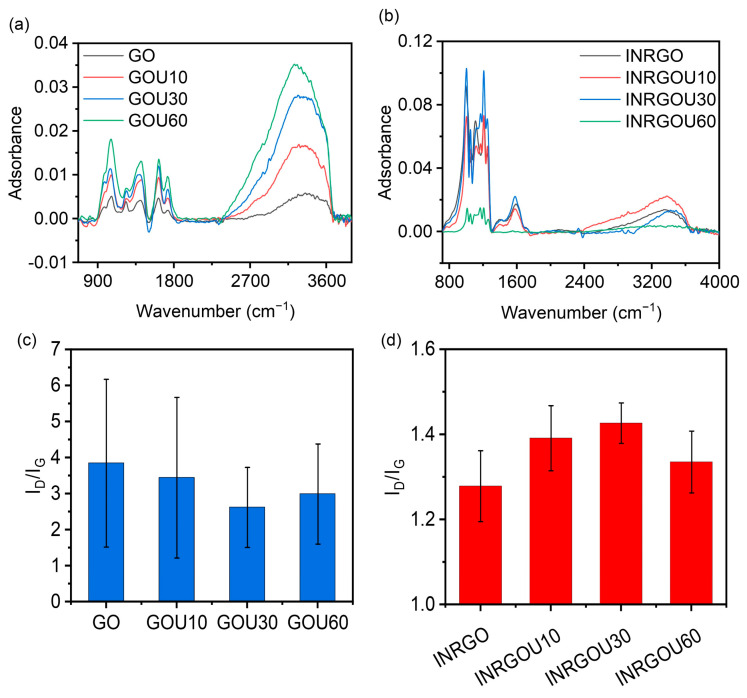
FTIR and Raman characterizations for the control group (CG) and experimental group (EG): FTIR spectra of the CG (**a**) and EG (**b**), and statistical histogram of the peak-area ratios (I_D_/I_G_) from Raman spectra of CG (**c**) and EG (**d**).

As described above, the MER (EG) had significantly enhanced GO adsorption compared with SER (CG). Among the EG, INRGOU60 had the best adsorption performance. To reveal the adsorption mechanism, we analyzed CG and EG samples with FTIR, Raman, and XPS. Figure 6a,b show the IR spectra of the CG and EG, respectively. The bands of CG at around 1725, 1729, 1727, and 1725 cm^−1^ are ascribed to the carbonyl group, and the bands at 3382, 3322, 3313, and 3237 cm^−1^ are to the hydroxyl group. It was found that the absorbance of the hydroxyl and carbonyl groups increased with the sonication time for CG [61,62,63]. The ratio of hydroxyl and carbonyl groups was semi-quantitatively calculated to accurately compare the changes of carbonyl groups for individual CG. The proportions of hydroxyl groups in GO, GOU10, GOU30, and GOU60 were 48.80%, 68.37%, 76.34%, and 76.90%, respectively, and the carbonyl group proportions were 27.2%, 5.39%, 6.36%, and 0.23%, respectively. The proportion of carbonyl groups in CG decreased gradually with the sonication time. Therefore, GO and GOU10 had the most minor proportion of hydroxyl groups, but their adsorption performances were better than others. Thus, other interactions like the multilayer structure from GOs probably had extra assistance. Figure 6b shows the FTIR spectrum of the EG. The bands at around 1722 cm^−1^ are ascribed to the carbonyl group, and the bands at about 3367, 3382, 3459, and 3359 cm^−1^ are to the hydroxyl group. Compared with the CG, the absorbance regarding the hydroxyl and carbonyl groups of EG decreased significantly. This result indicates a significant removal of oxygen-containing groups from the EG surface. The absorbance of the hydroxyl and carbonyl groups of INRGOU60 was the weakest. The spectrum of EG in 800~1300 cm^−1^ appeared with many miscellaneous peaks, possibly due to the presence of C-H groups. Its appearance corroborated the conversion of the carbonyl group to the hydroxyl group during the MER [30]. Figure 6c,d show the Raman results for CG and EG. The intensity ratio of the D and G bands (I_D_/I_G_) is widely used to evaluate the quality of carbon materials [64,65,66,67]. The statistical results are, in principle, closely related to the average distance between structural defects on the graphene basal plane, illustrating the disorder and defects of folds, edges, and pores [32]. The GOU30 had the lowest average I_D_/I_G_ ratio (2.62) in CG, and the INGOU30 of EG was the highest (1.43) in EG. The significantly lower ratios of EG indicate that the GO structure had been restored mainly of the sp^2^ carbon network. For the CG, because the I_D_/I_G_ ratios of GOU30 (2.62) and GOU60 (2.99) were lower than those of GO (3.85) and GOU10 (3.45), GOU30 and GOU60 had lower content of oxygen-containing groups on the surface than GO and GOU10. This difference somewhat explained that the adsorption capacity of GO (1806.67 mg/g at 283.15 K, 2025.19 mg/g at 298.15 K) and GOU10 (1518.52 mg/g at 298.15 K) was better than the others of the same group (Figure S3). For the EG, the INRGO (1.28) and INRGOU60 (1.34) had lower ratios than INRGOU10 (1.39) and INRGOU30 (1.43). Although the oxygen-containing groups on INRGO and INRGOU60 are assumed to be effectively removed, it is more reasonable to conclude the reduction for the INRGO was almost the same given small deviations. Therefore, the size-induced GO structural changes would mainly lead to the large variation of the adsorption capacity.

**Figure 7:**
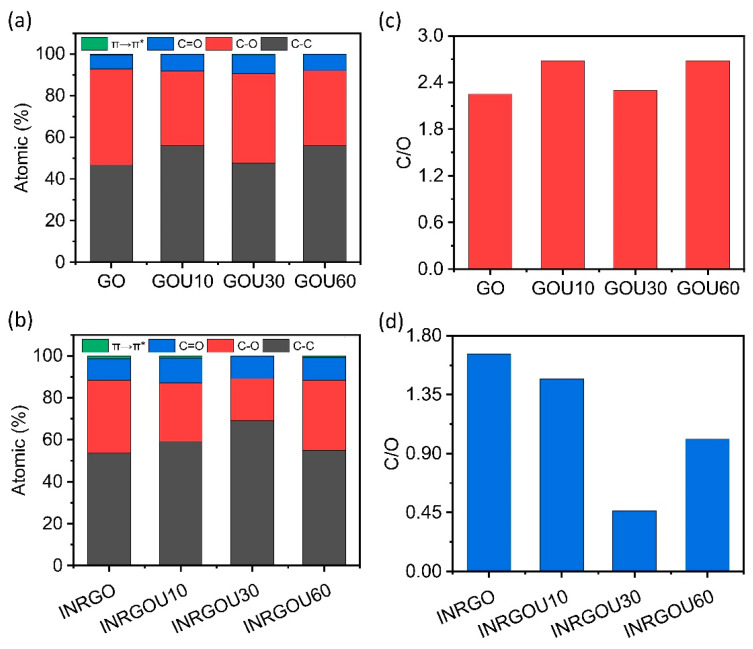
XPS analysis: composition of surface functional groups of the control group CG (**a**) and experimental group EG (**b**) and the C/O ratios for the CG (**c**) and EG (**d**).

Furthermore, the EG and CG XPS was also conducted to characterize the functional-group content (Figure 7) [68]. We obtained the ratios of element carbon to oxygen (C/O) for each GO from the broad spectra and four peaks of bonding from the C1s narrow spectra (not shown), including C-H/C-C at 284.8 eV, C-O at 286.5 eV, C=O at 288.5 eV, and π–π^⁎^ at 290.9 eV (Table S5).

Figure 7a,b show the proportion histograms of the CG and EG. The C-C (C-H) group accounted for 46.8%, 56.03%, 47.56%, and 55.93% for individual CG, and 53.82%, 59.28%, 69.19%, and 54.92% for EG, respectively. Compared with CG, the proportion of C-C (C-H) and C=O (O-C=O) groups increased significantly, but the C-O (C-OH/C-O-C) group decreased in EG. This change indicates that many oxygen-containing groups were removed from the EG after in situ treatment (MER). The proportion of the C-O (C-OH/C-O-C) group in CG was 45.95%, 35.72%, 43.02%, and 36.09%. Figure 7c shows that C/O values of GO, GOU10, GOU30, and GOU60 were at ~2.4—rather identical, despite different-time sonication. Additionally, many oxygen-containing groups of CG remained. As demonstrated, the adsorption capacity of GO and GOU10 was significantly better than GOU30 and GOU60, and they conformed to the multilayer adsorption process at low temperatures, consistent with previous studies [4,69,70,71]. Thus, the adsorbents with more multilayer structures would have the AO molecules entrapped between the interlayers, contributing more scored adsorption capacity. Thus, the adsorption behavior of CG was changed from multilayer adsorption to single-layer adsorption with the increase in temperature and sonication time.

The C/O ratios of EG were 1.66, 1.47, 0.46, and 1.01, respectively (Figure 7d and Table S5). The oxygen-containing moieties of the EG were considerably enriched after in situ treatment compared to the CG. Such a change must be an essential reason for changing the adsorption behavior of EG (Table S6).

The proportions of C-O (C-OH/C-O-C) groups in EG were 34.52%, 27.81%, 19.98%, and 33.36%, respectively (Figure 7b). These values were more significant than those of CG. It implied that a complex reaction might exist. As we reported previously [30], for example, the C=O proportion was less than before. However, this result does not agree. Instead, it somewhat increased. More interestingly, the C-O was ever considered more upon the MER [30], in contrast to the current shrinkage. Additionally, the C-C bonds were also recovered. Therefore, non-chemical progress, such as π–π interaction and Van der Waals forces, became dominant. The final adsorption behavior subsequently changed to the Freundlich multilayer model from the Langmuir monolayer.

**Figure 8:**
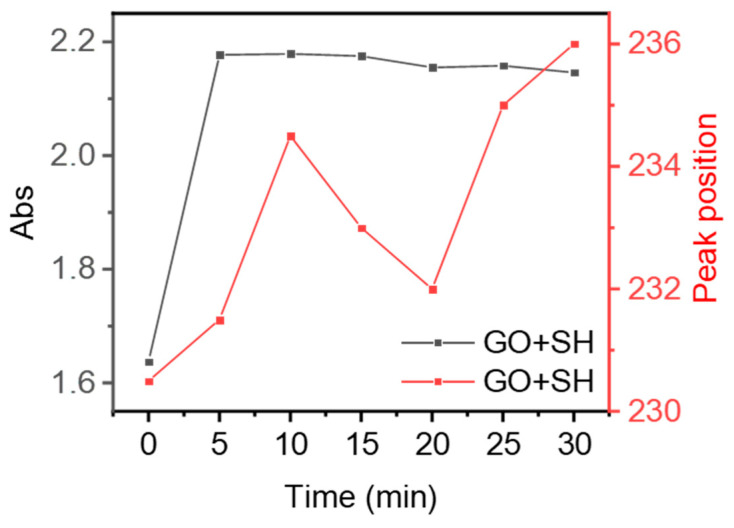
Changes of UV characteristic peak position and absorbance as a function of time after reduction treatment of GO.

Even though the above existed, a relatively large proportion of C-O and experimental adsorption capacity (4322.96 mg/g, Figure 6) occurred for the INRGOU60; many active adsorption sites were on the surface. Therefore, on the one hand, dehydration and condensation probably occurred between overly dense oxygen-containing groups during in situ treatment, which reduced the utilization rate of oxygen-containing groups, even though the EG (INRGOU30) had the most considerable oxygen content. On the other hand, the INRGOU30 had the lowest C/O (0.46), below the theoretical value (0.5~equivalent to CO_2_). However, the sizes of GOU30 and GOU60 were almost the same. Meanwhile, the adsorption capacity of INRGOU30 was not the maximum for the EG. Therefore, it was suspected that the extra oxygen resulted from the surface attaching many oxide impurities during the post-treatment. In the process of reduction, it is possible to adsorb reducing agents and lead to the increase of oxygen-containing groups on the surface of GO. The in situ reduction-treated GO still had a low C/O ratio (1.21) after washing, which also proved that reduction caused an increase in oxygen groups on its surface. In addition to the change of oxygen-containing groups, the GO undergoes a redshift after the reduction treatment, proving that the conjugated system of GO is repaired (Figure 8) [68]. This is also confirmed by the change in the absorbance of the UV characteristic absorption peak of GO, which increased from 1.638 to 2.177 after adding the reducing agent and finally leveled off [72]. In the UV spectrogram, a shoulder appears around 295 nm, which can be attributed to the π–π * transition of the aryl C=C bond and the n–π * transition of the C=O bond. The shoulder disappeared with increasing reduction time [73,74]. Effective reduction of GO should regenerate the C=C double bond in INRGO, thus enhancing the conjugation of π bonds. More research is being investigated to further the mechanism.

**Figure 9:**
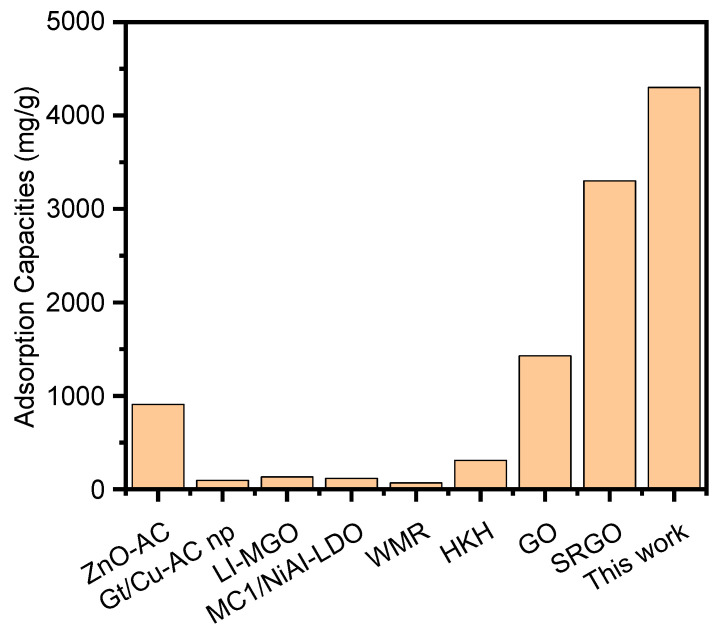
Research status of acridine orange removal based on adsorption in recent five years.(ZnO-AC: zinc oxide/almond shell activated carbon composite, Gt/Cu-AC np: green tea/copper-activated carbon nanoparticles, LI-MGO: Magnetic graphene oxide modified with 1-amine-3-methyl imidazole chloride ionic liquid, MCI/NiAi-LDO: NiAl layered double oxides modified magnetic corncob biochar, WMR: watermelon rinds, HKH: rice husk hydrochars, GO: graphene oxide, and SRGO: in-situ reduced graphene oxide).

The maximum adsorption capacity in this study was used to compare with that reported in previous studies (Figure 9). The adsorption capacity can reach 4.3 g/g—much higher than the adsorption results of other studies (Figure 9) [30,75,76,77,78,79]. Compared with other biomass materials and composite materials, the adsorption capacity of INRGOU60 in this study is much larger than these materials. The adsorption capacity of INRGOU60 is far superior to other adsorbents. Thus, the research on the adsorption of acridine orange dye in the past 5 years shows that the adsorption capacity of GO has been significantly improved after the successful use of ultrasound in the way of multi-equilibrium route adsorption in this study.

## 4. Materials and Methods

### 4.1. Reagents

Graphite of 325 mesh was purchased from Qingdao Huatai Lubricant Sealing S&T Co., Ltd., Qingdao, China. Concentrated sulfuric acid (98%) and sodium nitrate (AR) were purchased from the Beijing Institute of Chemical Reagents Co., Ltd., Beijing, China. Potassium permanganate (AR) was purchased from Beijing Yili Fine Chemicals Co., Ltd., Beijing, China. Deionized water, hydrogen peroxide solution, acridine orange (over 99.5%), and sodium nitrate were used as purchases without further treatment. For TEM and SEM tests, a SU9000 electron microscope from Hitachi was used (Beijing, China). Raman spectra were tested using a Horiba LabRAM HR Evolution model Raman spectrometer from Horiba (Kyoto, Japan). FTIR spectra were tested using the FTIR-650 model of Tianjin Gangdong Science and Technology Development Co., Ltd. (Tianjin, China). UV spectra were tested using a UV-visible spectrometer, TU-1901 model, from Beijing Purkinje general instrument CO., Ltd. (Beijing, China).

### 4.2. Preparation of GO

GO was prepared by the modified Hummers method [24,80]. In total, 5 g of graphite, 2.5 g of sodium nitrate, and 15 g of potassium permanganate were mixed thoroughly under ice bath conditions. A total of 110 mL of concentrated sulfuric acid was added to the mixture and stirred for 12 h. Afterward, the beaker was transferred to a constant-temperature water bath of 90 °C and stirred for 7 h. Then, 150 mL hydrogen peroxide was added, and the whole suspension was stirred for 30 min. The obtained solution was repeatedly centrifugated (9000–13,500 rpm) and diluted to remove the by-products until no precipitate occurred in the BaCl_2_ test. Afterward, the GO was stored in a fridge.

### 4.3. Preparation of GO and INRGO with Different Sizes

The concentration of as-prepared GO was determined through air drying. The GO with known concentration was divided into four equal parts. The first part was sonicated for 10 min, the second for 30 min, the third for 60 min, and the last without ultrasonication—recorded as GOU10, GOU30, GOU60, and GO, respectively. Following the same principle, all such different-size GOs reduced in situ at the 60th minute along the adsorption (MER adsorption) and were respectively defined as INRGOU10, INRGOU30, INRGOU60, and INRGO.

### 4.4. Adsorption Procedure

To facilitate research, we set up two groups on different occasions: the GO following the SER adsorption termed as the control group or CG, and the GO following the MER adsorption as the experimental group (EG). Therefore, the CG included GO, GOU10, GOU30, and GOU60, while the EG had INRGO, INRGOU10, INRGOU30, and INRGOU60. Moreover, the adsorption under different temperatures (283.15, 298.15, and 313.15 K) was also involved for the control and experimental groups. The GOs using the in situ reduction method were set as the experimental group (EG). The in situ reduction method consists of two parts. In the first stage, GOs (0.9 mg/mL, 0.2 mL) were added to the AO solution. At 60 min of the adsorption reaction, a Na_2_S_2_O_4_ solution (0.2 g/mL, 0.02 mL) was added. Meanwhile, Na_2_S_2_O_4_ had little effect on the structure of AO. In the second stage, the adsorption reaction was continued for 30 min. As for the influence of pH, the pH of Na_2_S_2_O_4_ in water was studied over time, and it was found that the pH value decreased from 5.67 to 4.24 at 2 min and then maintained at about 4.15 (Figure S2). The pH had little effect on the aqueous solution. Previous studies have compared GO, INRGO, and RGO (pre-reduced treated graphene oxide), among which INRGO has the best adsorption capacity [30]. Here, we mainly study the adsorption properties of different GO sizes in the MER adsorption mode to explore the GOs with the best adsorption performance in the MER mode.

### 4.5. Kinetics Experiment

Control group: under constant temperature, the GO solution (0.9 mg/mL, 0.2 mL) was added to the AO solution (100 mg/L, 20 mL). At 5, 10, 20, 30, 40, 50, and 60 min, a 1 mL disposable syringe with a filter head (pore size 0.45 μm) was used to purify the sampled suspensions for subsequent determination of concentration.

(1) $$\mathrm{Adsorption}\;\mathrm{capacity}=\frac{C_{0}{\;-\;C}_{t}}{m}\;\times \;V\;$$

where C_0_ (mg/L) and C_t_ (mg/L) are the initial concentration and the concentration at time t for AO, respectively, and V (mL) and m (g) indicate the total volume and mass value of adsorbents used, respectively.

Experimental group: under constant temperature, the GO solution (0.9 mg/mL, 0.2 mL) was added to the AO solution (100 mg/L, 20 mL). After 5, 10, 20, 30, 40, 50, and 60 min, 1 mL of solution was respectively collected with a syringe. Afterward, the Na_2_S_2_O_4_ solution (0.2 g/mL, 0.02 mL) was added, and the sampling continued to the 65th, 70th, 80th, and 90th minute.

Kinetic model fitting: kinetic experimental results require kinetic model fitting to study the adsorption behavior of GO. Two commonly used kinetic models are listed here [16]:

(2) $$\mathrm{Pseudo}-\mathrm{first}-\mathrm{order}\;\mathrm{model}:\;\frac{{\mathrm{dQ}}_{t}}{\mathrm{dt}}{=K}_{1}\left(Q_{t}{\;-\;Q}_{e}\right)\;$$

(3) $$\mathrm{Pseudo}-\mathrm{second}-\mathrm{order}\;\mathrm{model}:\;\frac{{\mathrm{dQ}}_{t}}{\mathrm{dt}}{=K}_{2}{\left(Q_{t}{\;-\;Q}_{e}\right)}^{2}\;$$

The formula shows that K_1_ and K_2_ represent the pseudo-first-order reaction rate constant (min^−1^) and pseudo-second-order (g mg^−1^min^−1^), respectively. Q_e_ (mg/g) stands for equilibrium adsorption.

### 4.6. Isotherm Experiment

Control group: under constant temperature, the GO solution (0.9 mg/mL, 0.2 mL) was added to 20 mL of AO solution of different initial concentrations (1, 5, 10, 25, 50, 100, and 200 mg/L). After 60 min of the reaction, the sample suspension was obtained by a disposable syringe, and then the sample was filtered through the filter head (pore size 0.45 μm) for subsequent concentration determination. Experimental group: the process was similar to the CG before the 60th minute. At the 60th minute, the Na_2_S_2_O_4_ solution (0.2 g/mL, 0.02 mL) was added. When the adsorption went on to the 90th minute, a 1 mL disposable syringe was used to filter through the filter head.

Isotherm model fitting: the equation for calculating the equilibrium adsorption capacity is as follows [81]:

(4) $$\mathrm{Adsorption}\;\mathrm{capacity}=\frac{C_{0\;}{-\;C}_{e}}{m}\;\times \;V$$

where C_0_ (mg/L) and C_e_ (mg/L) are the initial concentration and equilibrium concentration for AO, respectively, and V (mL) and m (g) indicate the total volume and mass value of adsorbents used, respectively.

Thermodynamic experimental results require kinetic model fitting to study the adsorption behavior of GO. Three commonly used kinetic models are listed here [55,82,83]:

(5) $$\mathrm{Langmuir}\;\mathrm{model}:\;\frac{Q_{e}}{Q_{m}}=\frac{C_{e}K_{L}}{C_{e}K_{L}+1}$$

(6) $$\mathrm{Freundlich}\;\mathrm{model}:\;Q_{e}{=K}_{F}C_{e}{}^{\frac{1}{n}}$$

(7) $$\mathrm{Tempkin}\;\mathrm{model}:\;Q_{e}=\mathrm{Bln}\left({\mathrm{AC}}_{e}\right);\;B=\frac{\mathrm{RT}}{b}$$

where Q_e_ stands for equilibrium adsorption capacity (mg/g), Q_m_ stands for theoretical maximum adsorption capacity (mg/g), C_e_ stands for equilibrium concentration (mg/L), K_L_ stands for the Langmuir equilibrium constant (L/mg), and K_F_ stands for the Freundlich equilibrium constant (L/mg). Additionally, 1/n represents the heterogeneity coefficient, b represents the Tempkin constant in terms of the heat of adsorption (KJ/mol), R represents the ideal gas constant, T represents the thermodynamic temperature, and A represents the binding constant related to the binding energy (L/mg); B represents the constant related to the heat of adsorption (KJ/mol).

## 5. Conclusions

The multiple-equilibrium-route-enhanced GO adsorption regarding sheet sizes was studied using acridine orange as the eliminating pollutant target. Different-size GOs were obtained via constant-temperature water-bath ultrasonication for varying times. We further investigated the size influences of GO in the SER adsorption and MER-enhanced adsorptions. Kinetic studies showed that all the SER GO adsorption of all temperatures correlated well with PFO and PSO kinetics, yet room temperature adsorption preferred the former. Meanwhile, we found that the isotherm adsorption behavior of GO changed with its size under SER but kept the same as the Freundlich model under MER. Additionally, we observed a tendency for capacity to increase from the global perspective as the size and temperature changed, although the individual GO varied from one to another because of the difference in thickness/layers and lateral size. Besides the original carbon and hydroxyl groups, interactions with the bonds/groups produced during the in situ liquid reduction played an essential role in the adsorption enhancement. However, many oxygen-containing groups may lead to dehydration and condensation between the GO surface groups, reducing the active adsorption sites on the GO surface. Size mattered for the MER-enhanced adsorption. As a result, a record adsorption capacity as high as ~4.3 g/g was obtained from the MER adsorption using 60-min sonication-tailored GO at 40 °C. We believe this work will push the development of the ultra-high-performance GO. Moreover, the novel MER continues to pave the way for environmental applications using GO as a highly effective adsorbent.

## Data Availability

Not applicable.

## Supplementary Materials

The following supporting information can be downloaded at: https://www.mdpi.com/article/10.3390/molecules28104179/s1, Table S1: Fitting parameters of the pseudo-first-order and pseudo-second-order models for the adsorption kinetics of CG and EG; Table S2: Linear fitting results regarding the capacity of different-size GO as a function of sonication time; Table S3: Fitting parameters of Langmuir, Freundlich, and Tempkin adsorption isotherms of CG; Table S4: Fitting parameters of Freundlich, Langmuir, and Tempkin adsorption isotherms of EG; Table S5: Deconvolution of C1s XPS spectra for individual CG and EG; Table S6: Isotherm adsorption behavior of the CG and EG at varying temperatures; Figure S1: pseudo-first-order and pseudo-second-order adsorption rate constants of CG; Figure S2: Effect of Na_2_S_2_O_4_ on the pH of aqueous solutions as a function of time; Figure S3: Statistics of the adsorption capacities of (a) CG and (b) EG at varying temperatures as a function of ultrasonication time.

## Sample Availability

Samples of the compounds are available from the authors.
