# Supplementary material for: Ultrasonication-Tailored Graphene Oxide of Varying Sizes in Multiple-Equilibrium-Route-Enhanced Adsorption for Aqueous Removal of Acridine Orange
Source: Molecules. 2023 May 18;28(10):4179. doi: 10.3390/molecules28104179 (PMC10223085; doi:10.3390/molecules28104179)
Supplement: Supplementary file 1 [file molecules-28-04179-s001.zip › molecules-2382386-supplementary.pdf]

Ultrasonication-Tailored Graphene Oxide of Varying Sizes in Multiple-Equilibrium-Route-Enhanced Adsorption for Aqueous Removal of Acridine Orange

Zhaoyang Han [1], Ling Sun [1,2,*], Yingying Chu [1], Jing Wang [1], Chenyu Wei [1], Yifang Liu [1], Qianlei Jiang [1], Changbao Han [1], Hui Yan [1] and Xuemei Song [1]

[1] Key Laboratory of Advanced Functional Materials, Institute of Advanced Energy Materials and Devices, Ministry of Education, Faculty of Materials and Manufacturing, Beijing University of Technology, Beijing 100124, China

[2] Beijing Guyue New Materials Research Institute, Beijing University of Technology, Beijing 100124, China

* Correspondence: sunling@bjut.edu.cn

Table S1. Fitting parameters of the pseudo-first-order and pseudo-second-order models for the adsorption kinetics of CG and EG

Temp /K	Samples	Experimental capacity (mg/g)	PFO			PSO		
			Q_e (mg/g)	K_1 (1/min)	R^2	Q_e (mg)	K_2 (g /mg·min)	R^2
283.15	GO	1024.44	959.73	0.45	0.9604	994.38	0.00114	0.9682
	GOU10	907.04	851.35	0.34	0.9832	891.72	0.000815	0.9933
	GOU30	948.89	920.58	1	0.9848	926.00	0.01	0.9823
	GOU60	1062.96	1024.07	0.42	0.9932	1009.78	0.01	0.9834
298.15	GO	1111.85	1126.70	0.42	0.9932	1104.00	0.00263	0.9868
	GOU10	1331.85	1252.01	0.36	0.9782	1200.00	0.00206	0.9654
	GOU30	1278.89	1287.00	0.43	0.9988	1263.00	0.00274	0.9911
	GOU60	1228.52	1202.96	0.57	0.9945	1192.00	0.00691	0.9921
313.15	GO	951.85	913.97	0.42	0.9851	941.38	0.00127	0.9891
	GOU10	807.41	792.62	0.62	0.9749	811.91	0.00243	0.9795
	GOU30	1193.33	1208.04	0.58	0.9931	1212.89	0.00411	0.9908
	GOU60	1107.04	1129.58	1.60	0.9839	1130.17	0.09849	0.9838
283.15	INRGO	2145.19	/	/	/	/	/	/
	INRGOU10	2054.81	/	/	/	/	/	/
	INRGOU30	3158.15	/	/	/	/	/	/
	INRGOU60	2320.00	/	/	/	/	/	/
298.15	INRGO	2152.22	/	/	/	/	/	/
	INRGOU10	2577.78	/	/	/	/	/	/
	INRGOU30	2284.81	/	/	/	/	/	/

	INRGOU60	3016.30	/	/	/	/	/	/
313.15	INRGO	1912.96	/	/	/	/	/	/
	INRGOU10	1920.86	/	/	/	/	/	/
	INRGOU30	2440.00	/	/	/	/	/	/
	INRGOU60	1858.52	/	/	/	/	/	/

Table S2. Linear fitting results regarding the capacity of different-size GO as a function of sonication time.

Parameters / Samples	Intercept		Slope		R^2
	**Value**	**Standard Error**	**Value**	**Standard Error**	
283.15 CG	953.31524	56.01518	+1.30069	1.6518	0.2367
298.15 CG	1218.50786	83.12502	+0.77079	2.45122	0.0471
313.15 CG	910.07833	117.50939	+4.19317	3.46516	0.422
283.15 EG	2270.26548	434.3065	+5.97088	12.807	0.09803
298.15 EG	2215.84476	204.86201	+11.67731	6.04105	0.6513
313.15 EG	2025.80762	247.04644	+0.2911	7.285	0.0007

Table S3. Fitting parameters of Langmuir, Freundlich, and Tempkin adsorption isotherms of CG.

Temp /K	Samples	Experimental capacity maximum (mg/g)	Freundlich			Langmuir			Tempkin		
			n	K_L (L/mg)	R^2	Q_m (mg/g)	K_L(L/mg)	R^2	B_T (KJ/ml)	A_T (L/mg)	R^2
283.15	GO	1806.67	4.68	699.96	0.95	1850.63	0.04	0.77	173.94	17.75	0.83
	GOU10	984.44	6.25	419.20	0.90	781.85	2.29	0.81	90.06	146.77	0.93
	GOU30	1066.67	7.07	580.12	0.74	1060.52	21.3	0.92	120.78	115.09	0.83
	GOU60	926.67	9.87	648.88	0.68	981.28	14.92	0.94	83.46	2895.16	0.77
298.15	GO	2025.19	8.95	1039.39	0.88	1768.83	9.92	0.87	143.74	2754.65	0.95
	GOU10	1518.52	8.49	806.89	0.87	1378.95	12.21	0.86	127.04	829.91	0.95
	GOU30	1727.41	8.53	685.66	0.76	1095.59	10.74	0.90	102.24	1089.06	0.83
	GOU60	1848.15	8.91	669.59	0.74	1047.14	13.71	0.90	93.74	1734.60	0.82
313.15	GO	897.04	8.96	559.64	0.79	899.37	2.25	0.95	76.38	2113.72	0.89
	GOU10	1644.44	9.30	652.32	0.86	975.16	64.51	0.93	77.58	9015.33	0.92
	GOU30	913.33	9.21	543.69	0.79	830.68	17.28	0.93	71.99	2640.40	0.86
	GOU60	1028.15	9.08	426.68	0.80	902.60	2.03	0.95	109.63	42.93	0.87

Table S4. Fitting parameters of Freundlich, Langmuir, and Tempkin adsorption isotherms of EG.

Temp /K	Samples	Maximum experimental capacity	Freundlich			Langmuir			Tempkin		
		(mg/g)	n	K_L (l/mg)	R^2	Q_m (mg/g)	K_L (l/mg)	R^2	B_T (KJ/ml)	A_T (l/mg)	R^2
283.15	INRGO	2191.11	5.18	769.32	0.97	1741.89	0.754	0.77	175.56	260.85	0.92
	INRGOU10	2808.15	5.12	984.43	0.93	2893.37	0.066	0.72	227.96	264.69	0.90
	INRGOU30	2745.93	4.32	796.60	0.97	2617.07	0.086	0.77	243.11	81.19	0.90
	INRGOU60	2316.30	5.09	839.15	0.97	2033.12	0.383	0.87	203.42	170.94	0.92
298.15	INRGO	2870.37	4.11	818.39	0.98	2732.53	0.114	0.86	203.42	170.94	0.95
	INRGOU10	2586.67	4.19	749.85	0.97	2546.25	0.088	0.83	267.91	65.47	0.92
	INRGOU30	3123.70	4.84	1022.25	0.96	3065.11	0.078	0.77	215.37	165.17	0.89
	INRGOU60	2940.00	3.36	641.30	0.99	2858.39	0.084	0.94	243.22	291.03	0.91
313.15	INRGO	2200.74	4.42	656.82	0.97	2063.88	0.105	0.82	169.04	266.21	0.88
	INRGOU10	2481.48	3.73	575.23	0.98	2316.59	0.071	0.87	213.98	51.62	0.88
	INRGOU30	2374.07	3.79	595.34	0.95	2422.29	0.059	0.82	328.84	15.73	0.81
	INRGOU60	4322.96	1.61	174.92	0.98	8495.96	0.006	0.95	328.84	15.73	0.58

Table S5. Deconvolution of C1s XPS spectra for individual CG and EG.

**Samples**	**C-C** (**284.8 eV**)	**C-O** (**286.5 eV**)	**C=O** (**288.5 eV**)	$\pi \rightarrow \pi^*$ (**290.9 ev**)
GO	46.8%	45.95%	6.84%	0.41%
GOU10	56.03%	35.72%	8.14%	0.11%
GOU30	47.56%	43.02%	8.87%	0.55%
GOU60	55.93%	36.09%	7.92%	0.06%
INRGO	53.82%	34.52%	10.41%	1.25%
INRGOU10	59.28%	27.81%	11.88%	1.02%
INRGOU30	69.19%	19.98%	10.71%	0.12%
INRGOU60	54.92%	33.36%	10.91%	0.81%

Table S6. Isotherm adsorption behavior of the CG and EG at varying temperatures.

Samples	283.15 K	298.15 K	313.15 K
GO	Freundlich	Tempkin	Langmuir
GOU10	Tempkin	Tempkin	Langmuir
GOU30	Langmuir	Langmuir	Langmuir
GOU60	Langmuir	Langmuir	Langmuir
INGGO	Freundlich	Freundlich	Freundlich
INRGOU10	Freundlich	Freundlich	Freundlich
INRGOU30	Freundlich	Freundlich	Freundlich
INRGOU60	Freundlich	Freundlich	Freundlich

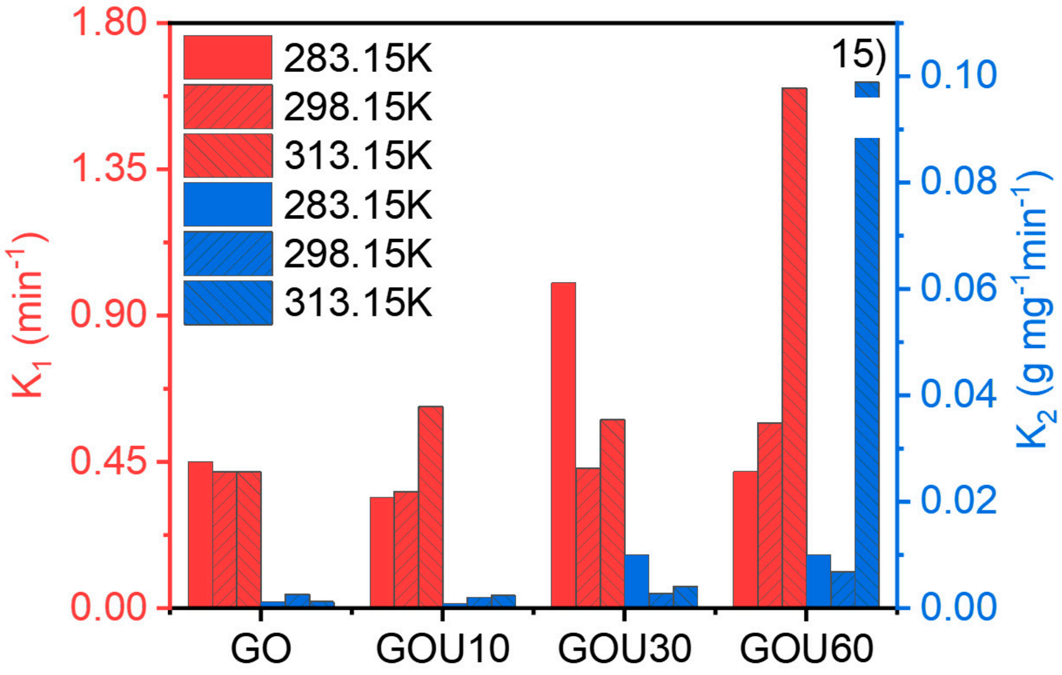

Figure S1. Comparison of pseudo-first-order and pseudo-second-order adsorption rate constants between control and experimental groups

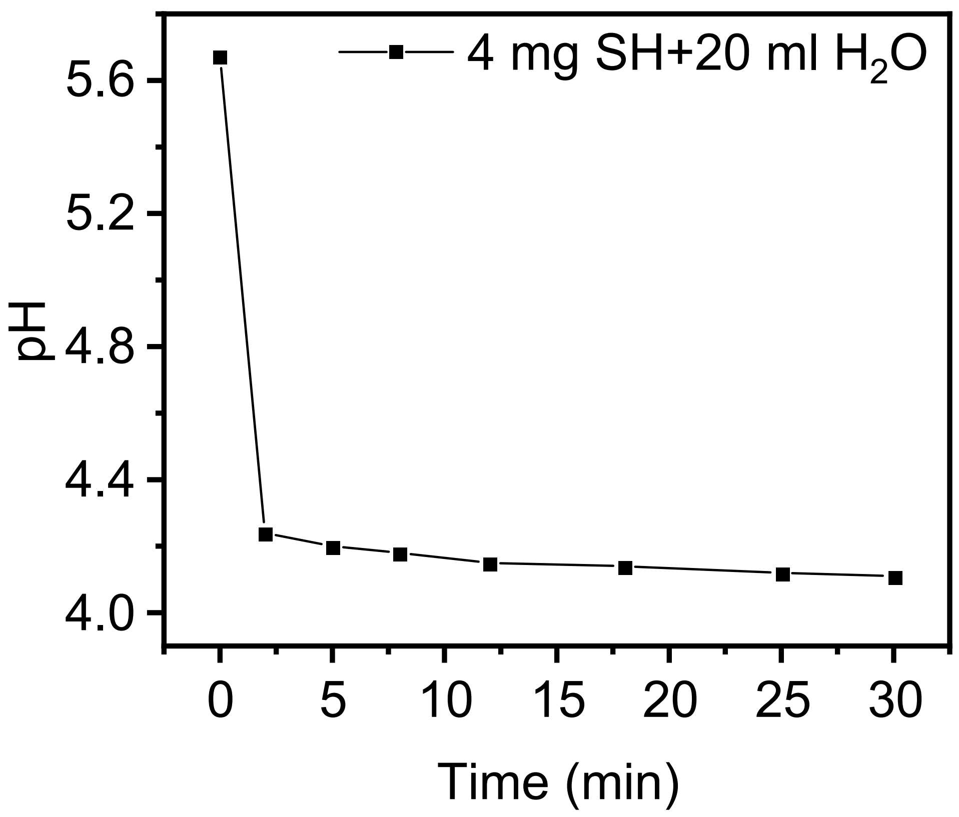

Figure S2. Effect of SH on the pH of aqueous solutions as a function of time

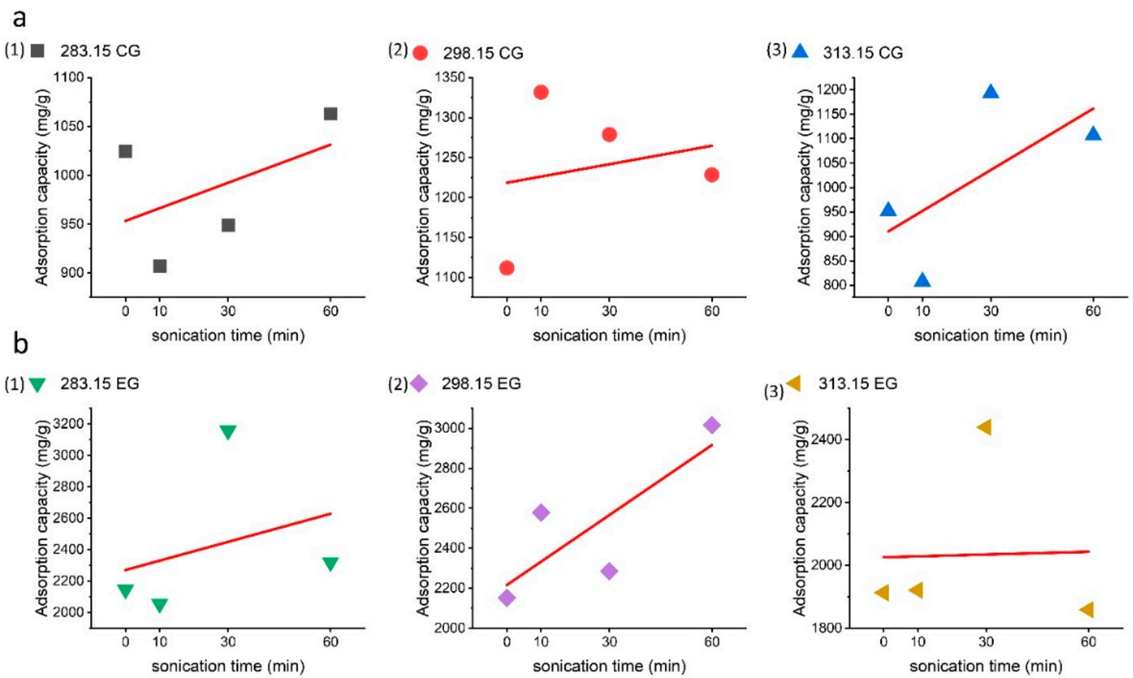

Figure S3. Statistics of the adsorption capacities of (**a**) CG and (**b**) EG at varying temperatures as a function of ultrasonication time. The kinetics adsorption capacity data collected from the experiments with the initial AO concentration at 100 mg/l were analyzed.
